# Unveiling the influence of salinity on bacterial microbiome assembly of halophytes and crops

**DOI:** 10.1186/s40793-024-00592-3

**Published:** 2024-07-18

**Authors:** Mohamed R. Abdelfadil, Sascha Patz, Steffen Kolb, Silke Ruppel

**Affiliations:** 1 Thaer-Institute, Faculty of Life Sciences, Humboldt University of Berlin, 10115 Berlin, Germany; 2 Department of Plant-Microbe Systems, Leibniz Institute of Vegetable and Ornamental Crops (IGZ), 14979 Großbeeren, Germany; 3 Microbial Biogeochemistry, RA Landscape Functioning, Leibniz Center for Agricultural Landscape Research (ZALF), Eberswalder Str. 84, D-15374 Müncheberg, Germany; 4 Computomics GmbH, Eisenbahnstraße 1, 72072 Tübingen, Baden-Württemberg, Germany

**Keywords:** Salinity stress, Microbiome structure, Halophytes, Non-halophytes, Meta-analysis, Core taxa

## Abstract

**Background:**

Climate change and anthropogenic activities intensify salinity stress impacting significantly on plant productivity and biodiversity in agroecosystems. There are naturally salt-tolerant plants (halophytes) that can grow and withstand such harsh conditions. Halophytes have evolved along with their associated microbiota to adapt to hypersaline environments. Identifying shared microbial taxa between halophyte species has rarely been investigated. We performed a comprehensive meta-analysis using the published bacterial 16S rRNA gene sequence datasets to untangle the rhizosphere microbiota structure of two halophyte groups and non-halophytes. We aimed for the identification of marker taxa of plants being adapted to a high salinity using three independent approaches.

**Results:**

Fifteen studies met the selection criteria for downstream analysis, consisting of 40 plants representing diverse halophyte and non-halophyte species. Microbiome structural analysis revealed distinct compositions for halophytes that face high salt concentrations in their rhizosphere compared to halophytes grown at low salt concentrations or from non-halophytes. For halophytes grown at high salt concentrations, we discovered three bacterial genera that were independently detected through the analysis of the core microbiome, key hub taxa by network analysis and random forest analysis. These genera were *Thalassospira*, *Erythrobacter*, and *Marinobacter*.

**Conclusions:**

Our meta-analysis revealed that salinity level is a critical factor in affecting the rhizosphere microbiome assembly of plants. Detecting marker taxa across high-halophytes may help to select Bacteria that might improve the salt tolerance of non-halophytic plants.

**Supplementary Information:**

The online version contains supplementary material available at 10.1186/s40793-024-00592-3.

## Background

Rapid environmental fluctuations of environmental factors by climate change and anthropogenic activities, increase stressors and affect biodiversity across different biomes [1, 2]. In particular, plant habitats are highly vulnerable to salinization, resulting in reduced plant fitness and productivity in agroecosystems [3–5]. There are naturally salt-tolerant plants (halophytes) on our planet that are adapted to saline habitats. These halophytes have evolved with their associated microbiome to develop adaptive salt tolerance and mitigate salinity stress [6, 7]. The plant-associated microbiome is considered a legacy assembled through the recruitment of precedent plants to adapt to the dynamics of environmental conditions [8–10].

Exploring and understanding the role of the microbiome of stressed plants is an opportunity to develop new strategies capable of rapidly improving plant adaptation to stresses. Various studies have elucidated the potential of microbial taxa isolated from plants that are naturally adapted to harsh environments to improve stress resistance in sensitive plants. For instance, inoculation of alfalfa with two bacterial taxa, *Halomonas* sp. and *Bacillus* sp., isolated from the rhizosphere of three halophyte species grown in high-saline soils, were able to improve alfalfa growth under salinity conditions of 1% NaCl [11]. Also, core microbiome members associated with the halophyte *Suaeda salsa* showed the ability to promote the growth of rice seedlings under salt stress [12]. Synthetic bacterial communities constructed from the core root microbiome of desert plants living under extreme weather conditions have exhibited the ability to induce salt tolerance in tomato plants by stimulating the activation of ionic homeostasis mechanisms [13]. Previous meta-analysis studies have described the capability of arbuscular mycorrhizal fungi enhancing the salinity tolerance of halophytes and non-halophytes [14, 15]. Although halophytes represent a repository of microbes that possess traits associated with salt tolerance of their host plants, they have not been in-depth studied comparatively with other non-halophytes using comprehensive microbiome datasets.

Hence, we performed a meta-analysis reanalyzing published 16S rRNA gene sequence datasets to reveal the impact of salinity on the structure of bacterial microbiomes and shared patterns between halophytes and non-halophytes. We used three independent data analysis approaches to verify our results, i.e., the core microbiome analysis, identification of key hub taxa by network analysis, and random forest analysis.

## Methods

### Microbiomes data acquisition and selection criteria

**Fig. 1:**
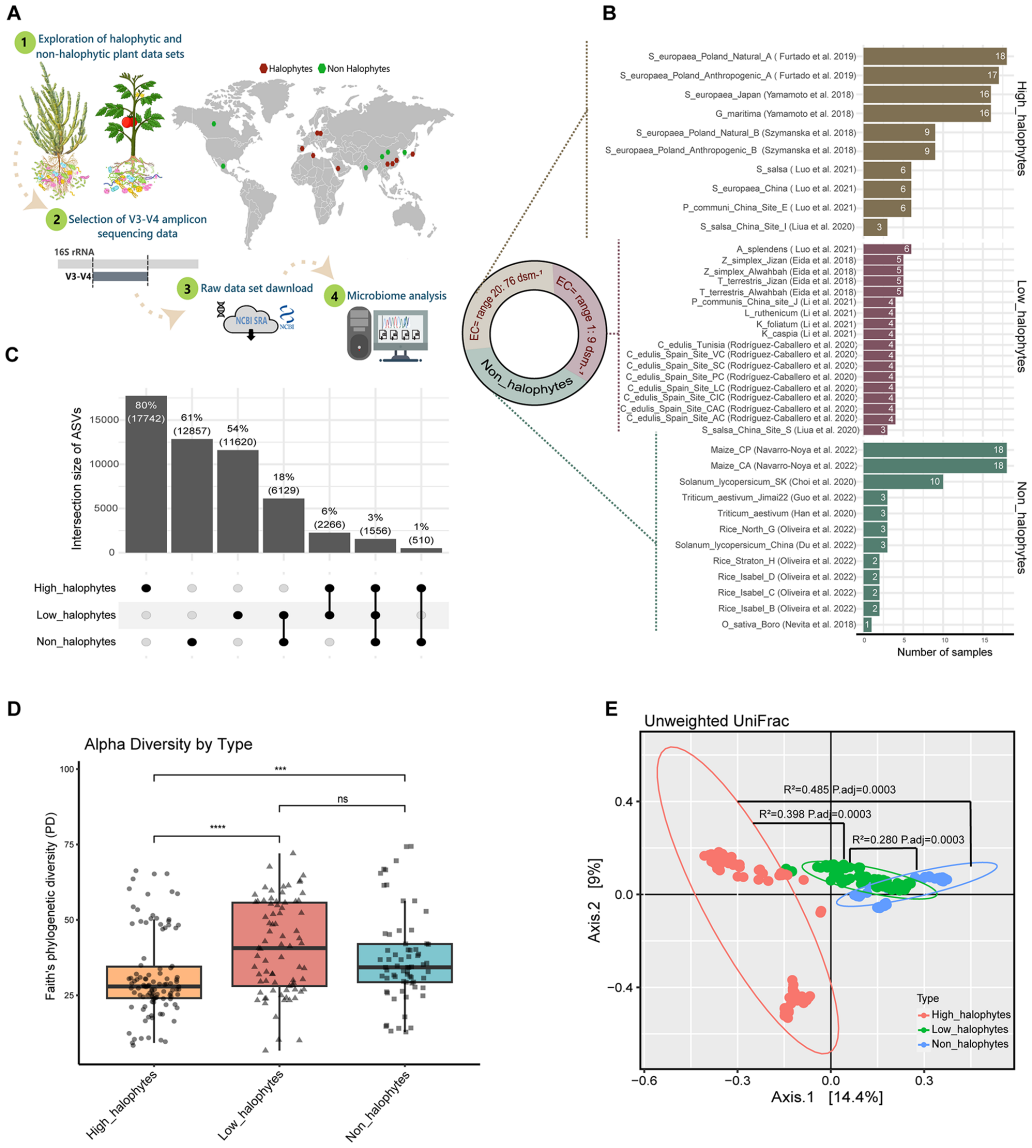
Differences between halophytic and non-halophytic plant microbiomes. (**A**) Schematic overview of an experimental analysis workflow. (**B**) Distribution of sample number size for each plant in a dataset from 15 diverse studies, encompassing high-halophyte, low-halophyte, and non-halophyte plants. (**C**) Upset plot showing the number of ASVs that were unique and shared between the high-halophyte, low-halophyte, and non-halophyte plants. (**D**) Alpha diversity, as evaluated using the PD index between high-halophyte, low-halophyte, and non-halophyte plants (**E**) Unweighted UniFrac revealed clustering between high-halophyte, low-halophyte, and non-halophyte plants

In our meta-analysis, we employed 16S rRNA gene amplicon sequence data from previously published studies conducted on various halophyte and non-halophyte rhizosphere microbiomes, as illustrated in Fig. 1A. To retrieve these studies, we performed systematic literature searches in Google Scholar until July 2022. We considered the entire root-associated microbiota and did not divide them into epiphytic and endophytic members since most available studies did not differentiate these two plant compartments. All studies included in our meta-analysis met the following selection criteria: (i) only those that had 16S rRNA sequence data (paired-end reads) generated via the Illumina sequencing (MiSeq) platform; (ii) those that targeted only the hypervariable region V3-V4 of the 16S rRNA gene. These selection criteria were adopted to minimize batch effects and potential biases during data analysis [16]. We utilized BioProject accession numbers from the selected studies to retrieve associated metadata information (e.g., plant parts, salinity concentration (EC values)), and accession numbers (Supplementary Table 1) [17–31]. For missing EC values in a couple of halophytic studies, we contacted the authors or searched the literature for relevant plants grew in the same salinity zone conditions to identify them. Afterwards, the raw sequencing data reads were downloaded from the NCBI Sequence Read Archive (SRA; https://www.ncbi.nlm.nih.gov/sra) via the NCBI SRA-Toolkit (https://github.com/ncbi/sra-tools).

### Studies selection and characteristics

16S rRNA gene sequence datasets of fifteen studies were analyzed because they met our selection criteria. These studies covered 40 plants with a total of 250 samples, encompassing eleven halophyte plant species (*Salicornia europaea*, *Suaeda salsa*, *Achnatherum splendens*, *Phragmites communis*, *Tribulus terrestris*, *Zygophyllum simplex*, *Carpobrotus edulis*, *Glaux maritima*, *Karelinia caspia*, *Lycium ruthenicum*, and *Kalidium foliatum*) and four non-halophyte plant species (*Solanum lycopersicum*, *Zea mays*, *Triticum aestivum*, and *Oryza sativa*) from 10 countries worldwide (Fig. 1A and B).

Plant samples were grouped based on related metadata parameters, such as plant species and salt concentrations in the rhizosphere, that consistently occurred in all studies. Halophytes can survive and reproduce in environments around 200 mM NaCl or more, which is nearly EC 20 dsm^− 1^ [32, 33]. Accordingly, this led to the emergence of three groups of plants: (i) ’High-halophytes‘ plants that grew under high salinity conditions > EC 20 dsm^− 1^; (ii) ’Low-halophytes‘ plants that grew under salinity conditions < EC 20 dsm^− 1^; (iii) ’Non-halophytes‘ crop plants (Fig. 1B).

### Fusion and analysis of 16S rRNA amplicon sequence datasets

Raw sequences from all studies were processed with one custom script using a combination of tools, consisting of USEARCH [34] and VSEARCH [35]. The pair-end reads of all samples were merged to create consensus sequences (38.5 M sequence, 83.63%) using USEARCH [34]. The merged sequences were quality filtered based on the maximum expected error (maxee = 1) and ± 10% of the expected sequence length using VSEARCH [35]. This step kept 29,101,927 sequences while discarding 9,423,481 sequences. Then, the filtered sequences were dereplicated by collapsing all identical sequences to identify the set of unique sequences via VSEARCH [35]. Subsequently, unique sequences were denoised to amplicon sequence variants (ASVs) with the UNOISE3 algorithm in VSEARCH [35], which controlled the ASV number to reduce the low-abundance noise of the dataset and achieve single-base ASV accuracy. Following that, VSEARCH [35] was utilized to detect and remove chimeras. As a result, 284,373 high-qualified amplicon sequence variants (ASVs) were obtained. In the next step towards generating the count table, all sequences were clustered at a 99% similarity cut-off value when mapping the merged sequences to ASVs by VSEARCH [35]. ASVs were taxonomically assigned using RDP classifier based on the RDP database with a confidence threshold of 0.8 via USEARCH [34, 36]. To construct the phylogeny for all ASVs, multiple sequence alignment was generated using MAFFT [37].

### Bacterial community analysis and subsequent statistics

All further downstream analysis were performed in R (version 4.3.0) based on the generated files, i.e., ASVs count table, taxonomy table, phylogenetic tree, and metadata file. The microbiome analysis was carried out using different R packages include: phyloseq [38], metagenomeSeq [39], vegan [40], microbiome [41], microViz [42], MicrobiotaProcess [43], microeco [44], metagMisc (www.github.com/vmikk/metagMisc), and picante [45]. ASVs assigned as chloroplasts and mitochondria were subsequently removed. Multiple filtering steps were applied to reduce the technical variability and mitigate sensitivity during structural composition and diversity analysis. These steps aimed for the exclusion of rare taxa through a set of criteria, as a taxon to pass in our analysis must be: (1) present in at least one sample (2) a minimum of 10 reads present in that sample (3) a minimum total read count equal 20 reads across all the samples. For alpha diversity analysis, samples were rarefied without replacement before estimating Faith’s phylogenetic diversity index (Faith’s PD) to adjust differences in library sizes across samples. The statistical significance of Faith’s phylogenetic diversity index was calculated for multiple comparisons using the Wilcoxon rank-sum test. Beta diversity was measured using unweighted UniFrac distance metrics and visualized by Principal Coordinates Analysis (PCoA) to describe the changes in microbiota composition across the plant groups utilizing functions from the phyloseq and ggplot2 packages in R. A PERMANOVA with adonis2 was performed on unweighted UniFrac distance metrics to assess the significant differences in overall microbiota composition and between plant groups using vegan and ecole packages in R. To represent differential abundances of taxa present between the plant groups, counts were normalized using the Total Sum Scaling TSS method followed by taxa filtering of low abundances (less than 0.0001). Heat trees were used to visualize the hierarchical structure of taxonomic differences while excluding any taxon less than 300 reads to address the most abundant taxa.

A random forest classification model was employed to identify the most important genera across the different plant groups. Besides, MeanDecreaseGini was selected as the indicator value to determine the importance of differentially expressed genera. For key hub taxa identification, a network analysis was constructed and visualized using NetCoMi [46]. The network associations were measured using the Semi-Parametric Rank-based approach (SPRING) method [47]. The taxa have agglomerated at the genus level, and the dataset filtered to include only the 170 taxa with the highest frequency of occurrence. The keystone taxa were detected as a node above the 95% quantile of the fitted log-normal distribution of the four normalized network metrics (degree, betweenness, closeness centrality, and eigenvector centrality). Core microbiomes were identified by stringent measures based on presence/absence patterns at the genus level across several different plant species associated with each of the three groups. Unique and shared of all core genera across the plant groups were visualized using the pheatmap package in R. To group the different plants based on their similar microbiota content, unsupervised hierarchical clustering with Ward D2 was constructed based on unweighted datasets (presence/absence genera) via stats package in R. All figures visualizations and data management were prepared using the following R packages: Metacoder [48], ggplot2 [49], UpSetR [50], pheatmap [51], and tidyverse [52].

## Results

### Differences in the microbiome structure between high-halophytes, low-halophytes, and non-halophytes

The unique and shared number of ASVs were detected to resolve the distribution of the ASVs between the plants in each group, i.e., high-halophytes, low-halophytes, and non-halophytes (Fig. 1C). We observed a high number of unique ASVs in the group of high-halophytes with 80% (17,742), followed by the non-halophytes with 61% (12,857) (Fig. 1C). The low-halophyte and non-halophyte groups shared the largest number of ASVs 18% (6,129), whereas only 6% (2,266) of all ASVs were shared between the plant groups high-halophytes and low-halophytes, and 3% (1,556) between all the groups (Fig. 1C).

We quantified the extent of microbiome diversity and variations in the compositions across the three groups of plants by determining the alpha diversity and beta diversity. Alpha diversity patterns, using phylogenetic diversity (Faith’s PD), revealed no significant differences between low-halophytes and non-halophytes (Wilcoxon test, *p* values > 0.05), while these two groups were significantly different from high-halophytes (Fig. 1D). For beta diversity, there were significant differences in the community structure between the three plant groups (*R*^*2*^ = 0.177, *p* = 0.0001, detected using PERMANOVA; permutation = 9999). Notably, principal coordinates analysis (PCoA) of unweighted UniFrac distance matrix revealed a clear separation of high-halophytes-associated microbiome composition distant from low- or non-halophytes (Fig. 1E). Hence, these analyses revealed that the low-halophytes plant group was closer to the non-halophytes group than the high-halophytes in terms of its microbiome compositions and diversity. The high-halophytes were represented by at least two distinct clusters of microbiomes. Removing the bottom cluster had only a minor impact on the most relative abundance plot and on the clustering of the plant species based on bacterial genus presence and absence (Figure S2 and S3).

### Common patterns in bacterial communities associated with different plant species

To what extent salinity level in the rhizosphere drives the specificity of microbiomes associated with plant roots was the next logical step in the analysis based on the aforementioned results.

**Fig. 2:**
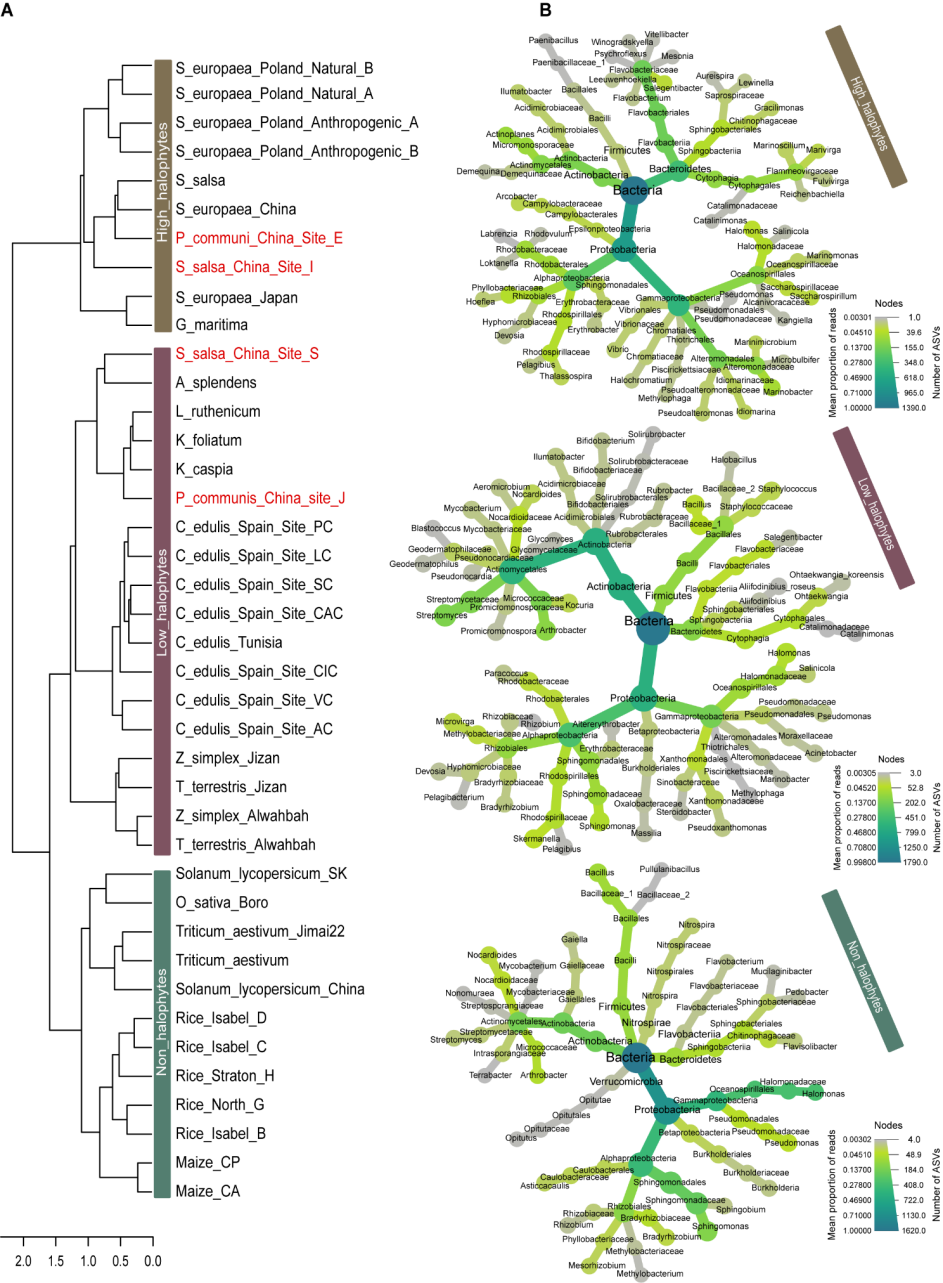
Salinity levels impact plant-associated microbiomes. (**A**) Dendrogram based on bacterial genus level of all plants included in the 15 microbiome studies. The red names present between two different clusters refer to the same plant species. (**B**) Heat trees represent the number of ASVs and the read abundance per taxonomic rank from phylum to genus for the high-halophytes, low-halophytes, and non-halophytes

Cluster analysis were applied to grouping all plants sharing similar microbiome compositions. A dendrogram of unsupervised hierarchical clustering of all 40 plants in our study revealed two main clusters (Fig. 2A). Interestingly, within the two main clusters, all the plants of the high-halophytes plant group were clustered together based on this analysis. The second cluster split into two sub-clusters, one comprising microbiome composition from the low-halophyte plants and the second from all the non-halophyte plants. In addition, this dendrogram illustrates that the salt concentrations in the rhizosphere have a big influence on the assembly of microbiome compositions around the plant root compared to the impact of plant species. Whereas, the same halophyte species diverged into two different clusters when grown at different salinity concentrations, i.e., *Suaeda salsa* (S_salsa_China_Site_I; EC value = 25 dsm^− 1^, and S_salsa_China_Site_S; EC value = 9 dsm^− 1^) or *Phragmites australis* (P_communis_China_Site_E; EC value = 31 dsm^− 1^, and P_communis_China_Site_J; EC value = 0.04 dsm^− 1^) (Fig. 2A).

Moreover, to give a thorough overview of the influence of salinity on the variation of taxa specificity, differential heat trees were used to identify taxa with higher abundances associated with plant groups (Fig. 2B). High differences in taxonomic structure occurred from phylum to genus level in the three plant groups, respective to their salinity concentration. The microbiome structure of high-halophytes group was uniquely enriched with halophilic microbial genera, e.g., *Marinobacter*, *Thalassospira*, *Actinoplanes*, *Marivirga*, *Salegentibacter*, and *Saccharospirillum* (Fig. 2B). In contrast, non-halophytes showed a richness in bacterial genera, such as *Sphingomonas*, *Pseudomonas*, *Bacillus*, *Halomonas*, *Bradyrhizobium*, *Arthrobacter*, which are the most common and abundant taxa across the crop plants (Fig. 2B). Likewise, low-halophytes resembled the non-halophytes in the high-abundant genera, e.g., *Streptomyces*, *Bacillus*, *Halomonas*, *Sphingomonas*, *Microvirga*, and *Arthrobacter* (Fig. 2B).

### Identification of marker taxa associated with high-halophytic, low-halophytic, and non-halophytic plants

We used three approaches to identify taxa (genus) that have a potential role in their groups, i.e., the core microbiome, microbiome-based feature selection, and key hub taxa (Table 1).

**Table 1:** On the bacterial genus level, three approaches were applied: (1) core taxa, (2) key hub taxa (network analysis), and (3) most important taxa (random forest). Shared genera between all three approaches are in bold within each group

Groups	(1) Core taxa	(2) Key Hub taxa	(3) Most important taxa
**High-halophytes**			
	***Thalassospira***	***Thalassospira***	***Thalassospira***
	***Erythrobacter***	***Erythrobacter***	***Erythrobacter***
	***Marinobacter***	***Marinobacter***	***Marinobacter***
	*Marivirga*	*Gracilimonas*	*Marivirga*
	*Devosia*	*Planomicrobium*	*Hoeflea*
	*Lewinella*	*Psychroflexus*	*Marinoscillum*
	*Marinoscillum*	*Martelella*	*Marinomonas*
	*Fulvivirga*	*Fulvivirga*	*Devosia*
	*Ilumatobacter*	*Tangfeifania*	*Lewinella*
	*Maritalea*		
	*Muricauda*		
	*Roseovarius*		
	*Altererythrobacter*		
	*Pelagibius*		
	*Halomonas*		
	*Loktanella*		
	*Roseivivax*		
**Low-halophytes**			
	***Kocuria***	***Kocuria***	***Kocuria*** *-* ***Rubellimicrobium***
	***Rubellimicrobium***	***Rubellimicrobium***	*Actinophytocola - Microvirga*
	*Microvirga*	*Povalibacter*	*Promicromonospora - Massilia*
	*Brevundimonas*	*Pontibacter*	*Skermanella - Domibacillus*
	*Skermanella*	*Steroidobacter*	*Glycomyces - Streptomyces*
	*Mycobacterium*	*Gemmatimonas*	*Ensifer - Ohtaekwangia_koreensis*
	*Sphingomonas*	*Agromyces*	*Steroidobacter - Planomicrobium*
	*Nocardioides*	*Nonomuraea*	*Nocardioides - Paracoccus*
	*Devosia*	*Lysinibacillus*	*Paenibacillus - Pseudonocardia*
	*Rhizobium*		*Solirubrobacter - Lysobacter*
	*Actinophytocola*		*Adhaeribacter - Saccharothrix*
	*Bacillus*		
	*Phenylobacterium*		
	*Pseudomonas*		
**Non-halophytes**			
	*Sphingomonas - Bradyrhizobium*	*Ignavibacterium*	*Sphingomonas - Bradyrhizobium*
	*Mesorhizobium - Bosea*	*Melioribacter*	*Mesorhizobium - Opitutus*
	*Opitutus - Paenibacillus*	*Syntrophobacter*	*Phenylobacterium - Nitrospira*
	*Novosphingobium - Phenylobacterium*	*Thiobacillus*	*Gemmatimonas - Flavisolibacter*
	*Roseomonas - Gemmatimonas*	*Rhodomicrobium*	*Pedobacter - Bacillus*
	*Flavisolibacter - Amycolatopsis*	*Methylobacter*	*Mucilaginibacter - Burkholderia*
	*Geodermatophilus - Nitrospira*	*Desulfosporosinus*	*Parafilimonas - Caulobacter*
	*Clostridium_sensu_stricto - Pedobacter*	*Methylocystis*	*Sphingobium - Angustibacter*
	*Nocardioides - Hyphomicrobium*	*Oligoflexus*	*Labrys - Reyranella_massiliensis* *Niastella*
	*Flavobacterium - Mycobacterium*		
	*Labilithrix - Kribbella*		
	*Gaiella - Chryseolinea_serpens*		
	*Rhodococcus - Pseudonocardia*		
	*Devosia - Microvirga*		
	*Flavitalea*		

**Fig. 3:**
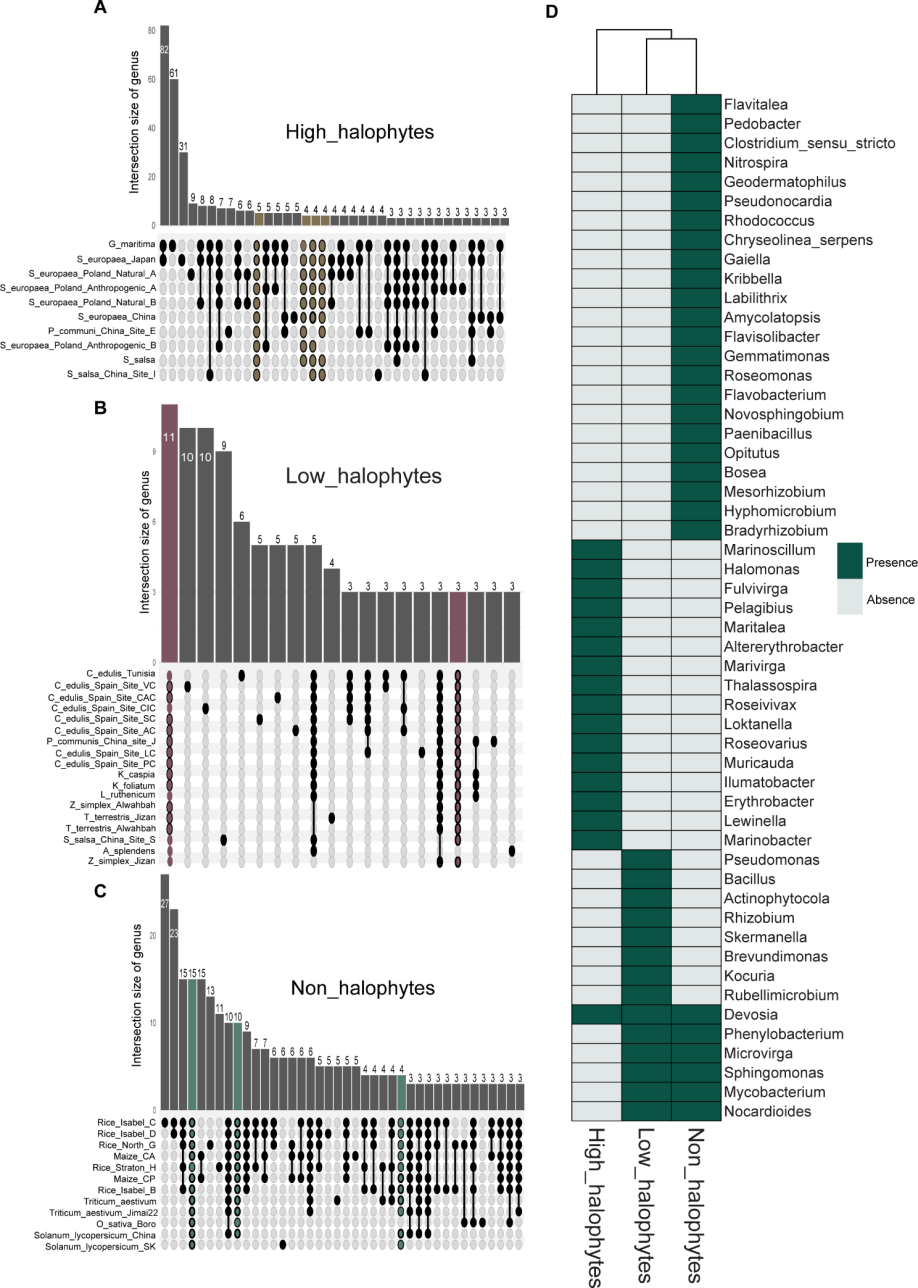
Core bacterial genera between the high-halophytes, low-halophytes, and non-halophytes. Upset plots displaying the unique and shared bacterial genera across all the plants in each group of high-halophyte (**A**), low-halophyte (**B**), and (**C**) non-halophyte plants. Color paths in A, B, and C indicate shared bacterial genera present between the plants. (**D**) Heatmap illustrating the unique and shared of all core genera across the high-halophytes, low-halophytes, and non-halophytes

The core taxa associated with each of the three plant groups were detected by their presence in all plants in each plant group or at least missing in only one plant of all plants of that group. In addition, we depicted the abundance distributions of detected core genera across each group and the overlap of all detected core genera across the three plant groups (Figure S1). Seventeen bacterial genera were shared between all high-halophyte plants (Fig. 3A). Notably, these 17 core genera were unique and not detectable in the core microbiome of low- or non-halophytic plants (Fig. 3D). Only, the genus *Devosia* occurred in the core microbiomes of all three plant groups (Fig. 3D). Core genera, such as *Ilumatobacter*, *Marivirga*, *Marinoscillum*, *Marinobacter*, and *Thalassospira*, were highly abundant in the microbiomes of high-halophytic plants (Figure S1A). Low-halophytes and non-halophytes were characterized by the presence of 14 and 29 core bacterial genera, respectively (Fig. 3B and C). In contrast to halophytes, approximately 50% of the core genera detected in low-halophytic plants also occurred in the core microbiome of non-halophytes, e.g., *Nocardioides*, *Sphingomonas*, *Phenylobacterium*, *Microvirga*, *Mycobacterium*, and *Devosia* (Fig. 3D).

**Fig. 4:**
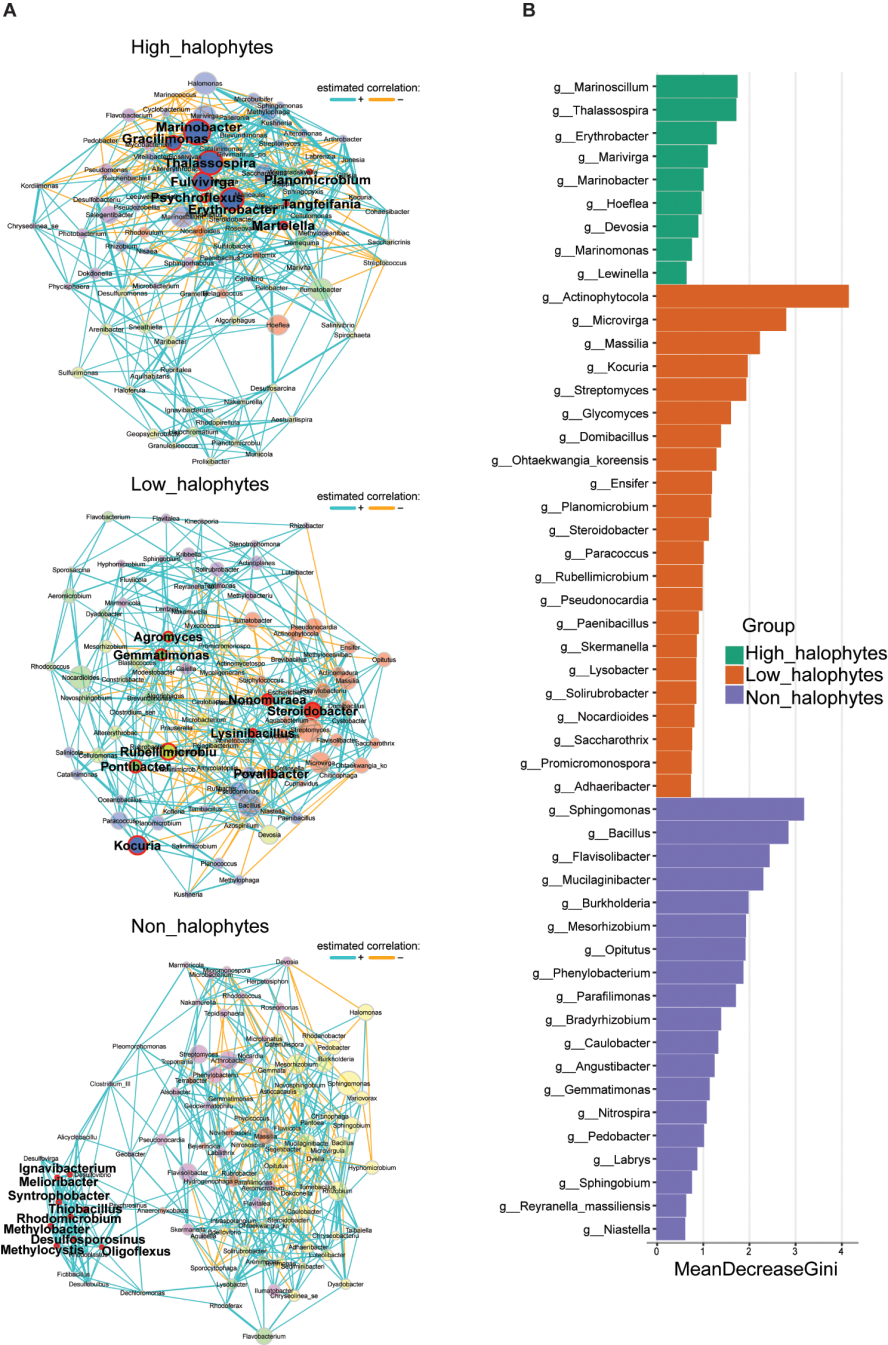
Most significant associated bacterial taxa linked to high-halophyte, low-halophyte, and non-halophyte plants. (**A**) Microbial co-occurrence networks of the highest frequency (170). Nodes represent bacterial genera, and different colors indicate different clusters determined by the fast greedy algorithm. The keystone taxa were detected as a node above the 95% quantile of the fitted log-normal distribution of the four normalized network metrics (degree, betweenness, closeness centrality, and eigenvector centrality). (**B**) Top 50 important genera identified using the supervised machine learning algorithm (random forest). Statistical significance of selected bacterial genera was assessed by Kruskal-Wallis test

Co-occurrence networks were conducted to identify highly interconnected taxa (hub genera) that influence the structure of microbiomes of the three plant groups (Fig. 4A). The networks between the three groups displayed a difference in their topologies and the sets of network hubs between the groups. Within the size of the constant component (170 taxa with the highest frequency) of co-occurrence, the positive edge percentages were almost similar for non-halophytes and low-halophytes (83%), while they were lower for high-halophytes (78%). We assessed the network’s different centrality measures using degree, betweenness, closeness centrality, and eigenvector centrality to determine key hub taxa (which are central to maintaining the stability of the community) for the co-occurrence networks of each plant group. Nine hub bacterial genera were identified for each group (Fig. 4A). The hub taxa in the high-halophytes network included the genera *Erythrobacter*, *Fulvivirga*, *Gracilimonas*, *Marinobacter*, *Martelella*, *Planomicrobium*, *Psychroflexus*, *Tangfeifania*, and *Thalassospira*. The genera *Agromyces*, *Gemmatimonas*, *Kocuria*, *Lysinibacillus*, *Nonomuraea*, *Pontibacter*, *Povalibacter*, *Rubellimicrobium*, and *Steroidobacter* were hub genera in the low-halophytes. Non-halophytes hub genera comprised *Desulfosporosinus*, *Ignavibacterium*, *Melioribacter*, *Methylobacter*, *Methylocystis*, *Oligoflexus*, *Rhodomicrobium*, *Syntrophobacter*, and *Thiobacillus*. In the high-halophytes plant group four genera, namely *Thalassospira*, *Marinobacter*, *Erythrobacter*, and *Fulvivirga*, were also observed in the set of core taxa, (Table 1).

At the genus level, we utilized a supervised machine learning algorithm (random forest) as the feature estimator to determine the top 50 taxa that were ranked for all three plant groups (Fig. 4B). Nine bacterial genera were identified as the most important in the high-halophytes. Out of these, the genera *Marinoscillum*, *Thalassospira*, *Erythrobacter*, and *Marinobacter* showed the highest values in the group. Unexpectedly, except for *Hoeflea*, all detected genera occurred as well as core taxa of high-halophytes (Table 1). While the genera *Actinophytocola*, *Microvirga*, *Massilia*, and *Kocuria* were the highest among the 22 most important genera detected in the low-halophytes. The highest important genera associated with non-halophytes were *Sphingomonas*, *Bacillus*, *Flavisolibacter*, and *Mucilaginibacter*. We observed that 6 and 9 genera occurred in core microbiomes of low- and non-halophytic plants, respectively (Table 1). Across all analytical approaches, three marker genera were commonly observed for high-halophytes: *Thalassospira*, *Erythrobacter*, and *Marinobacter* (Table 1). Nonetheless, we consider bacterial genera that were detected by at least two approaches as putative marker taxa, i.e., the genera *Marinoscillum* and *Fulvivirga* (Table 1).

## Discussion

Microorganisms cover a maximal broad range of ecological niches and can thrive under conditions that are deleterious for most multicellular organisms. Since they often have a sessile lifestyle, they have developed many traits that allow them to withstand a broad range of abiotic stresses, such as salinity [53, 54]. Microorganisms are considered the earliest life forms on earth and co-exist and co-evolve with their multicellular hosts, such as plants, in facing extreme environmental conditions [55, 56]. Interactions between the microbiome and its host plants that jointly adapt to different abiotic stresses are an ecological strategy of plant holobionts to shorten adaptation time and to improve holobiont’s resilience to dynamic stress conditions [57, 58]. Our meta-analysis has elucidated that changes in salinity levels drive the locally adapted rhizosphere microbiome structure of similar and different host plant species. A clear difference in alpha diversity and microbiome composition was observed for halophyte plants facing high salinity concentrations in their rhizosphere compared to halophytes that grew under low salinity or non-halophytic plants.

Previous studies have demonstrated the direct and indirect effects of abiotic stresses on the response of plant-associated and locally adapted microbiomes. One previous study has suggested that the abiotic factors mainly impact on the diversity of the rhizosphere bacterial microbiome associated with the halophytes and xerophytes in arid environments [59]. Differences in salinity around the same *Phragmites australis* species affected the structure of microbiome of the rhizosphere [60]. Salinity has been proven a determinant of microbiome structure of the rhizosphere of two date palm (*Phoenix dactylifera*) cultivars [61]. Habitat fragmentation also leads to significant shifts in the diversity and composition of microbiomes associated with plants [62]. Differences in the microbiome composition of two genotypes of *Arabidopsis thaliana* that grew at two different geographical sites have been observed [63]. Harsh environmental conditions, such as flooding, can significantly alter the composition and structure of rhizosphere microbiota of cereal crop plants (*Triticum aestivum* L.) [64]. Changes in the *Populus*-microbiome assembly are dynamic over the season, and seasonal factors are responsible for these shifts of microbiome composition [65].

Collectively, these results indicate that plant microbiome assembly depends substantially on the abiotic stresses and environmental factors, and is not only dependent on the host plant species. Thus, abiotic factors can strongly affect plant bacterial microbiomes and may explain that the microbiomes of the low-halophytes when grown at low levels of salt, were more similar to those of non-halophyte plants than to microbiomes of the high-halophytes. (Figures 1 and 2). Eventually, these conclusions explain why the halophytes of the same plant species can assemble different bacterial genera when grown under different salinity levels (Fig. 2A).

Salinity in the rhizosphere is a crucial factor driving diversification within the bacterial microbiome between halophytic and non-halophytic plants. Although the high-halophytes and non-halophytes share according to our meta-analysis even a same bacterial family, i.e., Phyllobacteriaceae. This bacterial family has branched completely into different genera that were either enriched in high-halophytes or non-halophytes, i.e., *Hoeflea* and *Mesorhizobium*, respectively (Fig. 2B). The genus *Hoeflea* is often associated with saline environments [66, 67], while the genus *Mesorhizobium* is regularly associated with non-halophytic crops [68]. When plants are exposed to salt stress, they are capable of recruiting specific bacterial microbiomes that support the mitigation of salinity stress, regardless of the salinity tolerance capacities of these plants [69]. Hence, identifying the plant microbiome members that are adapted to a particular abiotic stressors provides an opportunity to improve the adaption and resilience of the susceptible plant species to that specific stressor. There remains a challenge of identifying the microbiota members associated with improved stress tolerance [70]. Accordingly, we identified taxa associated with plants that can cope with salinity stress and that may represent microbiome legacy for these halophytes and may be missing in non-halophytic plants, such as many crops.

Such salinity-specific microbial taxa were detected based on their presence across a comprehensive collection of salt-tolerant plants belonging to the same and different plant species and grown under the same conditions and salinity levels. We found 17 common bacterial genera across all high-halophytes. With the exception of one genus, all other 16 genera were halophilic and were not detected in other cores of low- and non-halophytic holobionts (Fig. 3D). Four of these halophyte-associated genera were part of 8 core taxa also detected in the mangrove trees, which thrive under high-salt concentrations, and these core taxa suggest that they probably have the potential functions to promote host survival and resilience [71]. Interestingly, five genera of high-halophytes core microbiome detected in this meta-analysis, i.e., *Thalassospira*, *Erythrobacter*, *Fulvivirga*, *Marinoscillum*, and *Pelagibius*, have also been previously identified as shared genera between the domesticated and wildtype microbiome of the halophyte *Salicornia europaea* [72].

Three genera of the high-halophytes group, i.e., *Thalassospira*, *Erythrobacter*, and *Marinobacter* are halophilic bacteria and were commonly detected through all the three used analytical approaches (Table 1). Therefore, we consider these taxa as potential marker taxa and possibly they contributed to salt stress tolerance. These three halophilic genera have been isolated from various halophytes’ roots that grew under high salt conditions [73–75].

Although few studies have evaluated the functions of isolates of these genera, i.e., *Thalassospira*, *Erythrobacter*, and *Marinobacter*, they have shown some promising traits for promoting plant growth and improving salinity tolerance. For instance, *acdS* gene was detected in the *Thalassospira* sp. isolated from the rhizospheres of *Salicornia europaea* and *Aster tripolium* grown under high salinity conditions [74, 76]. In addition, *Thalassospira* sp. isolated from *Salicornia ramosissima* contained plant-growth promotion traits such as phosphate solubilization and indole-3-acetic acid (IAA) production [77]. Furthermore, *Marinobacter* sp. isolated from *Allenrolfea vaginata* can promote the growth of *Arabidopsis thaliana* under salt-stress [78]. And one study evaluated *Erythrobacter* sp. isolated from marine sediments and reported that the isolate contained genes required for siderophore production and enzymes for phosphate solubilization [79].

Although we tried to collect all relevant studies with high-quality data in our meta-analysis, we faced a challenge due to the limited availability of datasets and metadata that inform about the environmental conditions. Insufficient availability of datasets and metadata descriptions from studies led to uneven sample sizes across different plant groups. One key challenge was that we could not include other ecological factors’ impact, i.e., soil chemical and physical parameters and their interactions on the rhizosphere microbiome. With these limitations, we focused only on electrical conductivity (EC) values as the indicator of soil salinity. However, including data from different locations worldwide, we were able to investigate the salinity impact between different halophytes and non-halophytes.

In our meta-analysis, we investigated only the bacterial microbiome as it has been more often studied and more is known about the functional role in halophyte plants compared to fungi and Archaea. Future studies and in vitro experiments are still needed to explore and validate the halophyte microbiomes marker genera. Also, the use of alternative next-generation sequencing platforms in future would allow for a higher resolved taxonomic resolution of the halotolerant plant microbiome.

## Conclusions

Our findings demonstrated that salinity is one of the key environmental factors controlling the structure of the rhizosphere microbiome. We identified specific bacterial genera that likely mitigate salinity stress in plants and have collected evidence that these genera occur in several different plant species worldwide. The study results reinforce the relevance of “cry-for-help” theory of how stressed plants possibly assemble specialized microbiomes to mitigate abiotic stresses [80]. Our meta-analysis has identified bacterial marker taxa across high-halophytes that are relevant to improve the salt tolerance of non-halophytic plants.

### Electronic supplementary material

Below is the link to the electronic supplementary material.

Supplementary Material 1

Supplementary Material 2

## Data Availability

No datasets were generated or analysed during the current study.
