# Supplementary material for: Unveiling the influence of salinity on bacterial microbiome assembly of halophytes and crops
Source: Environ Microbiome. 2024 Jul 18;19:49. doi: 10.1186/s40793-024-00592-3 (PMC11256479; doi:10.1186/s40793-024-00592-3)
Supplement: Supplementary file 1 — Supplementary Material 1 [file 40793_2024_592_MOESM1_ESM.docx]

**Supplementary Figures**


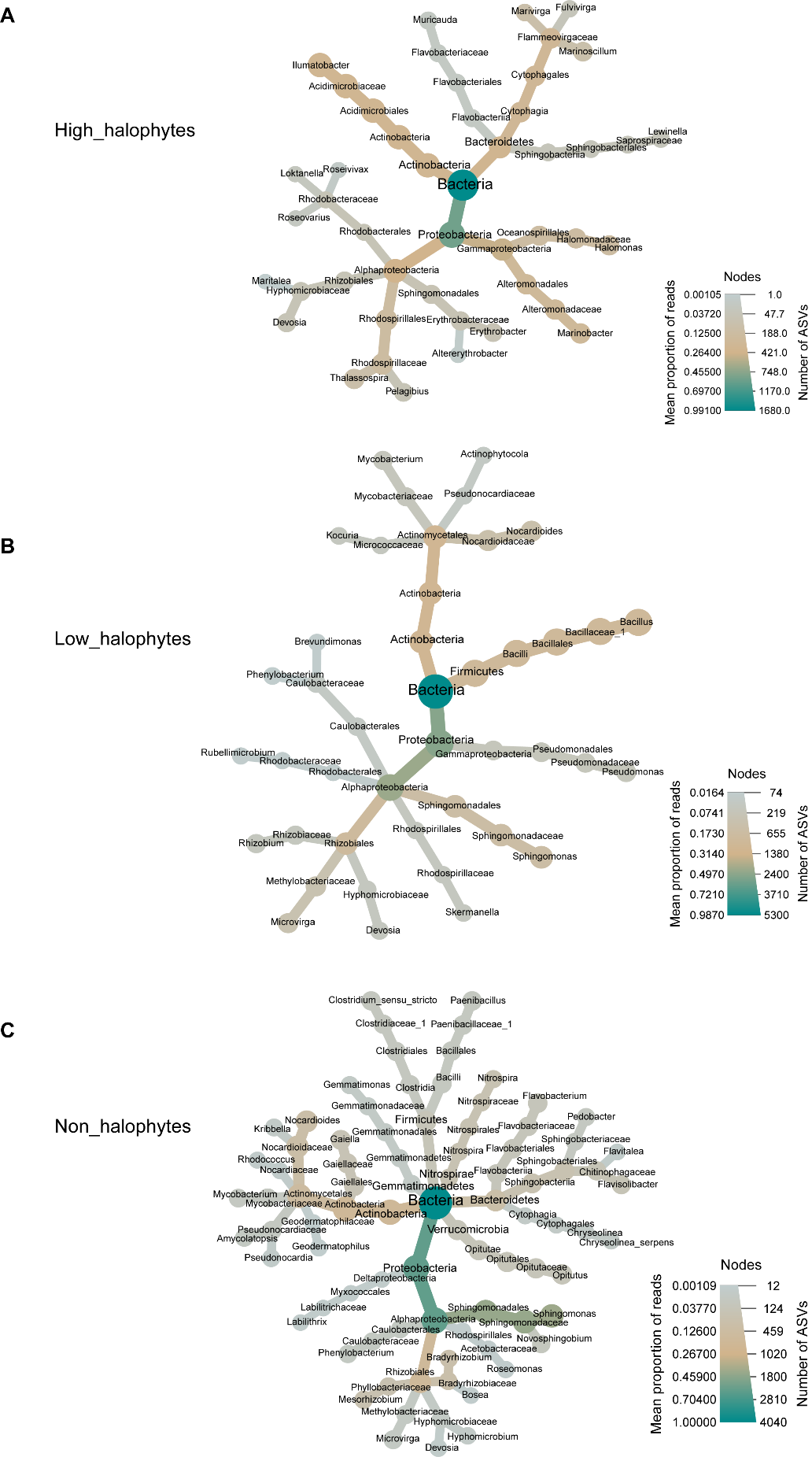


**Figure S1**. Heat trees represent the abundances of the core bacterial genera associated with high-halophytes, low-halophytes, and non-halophytes.


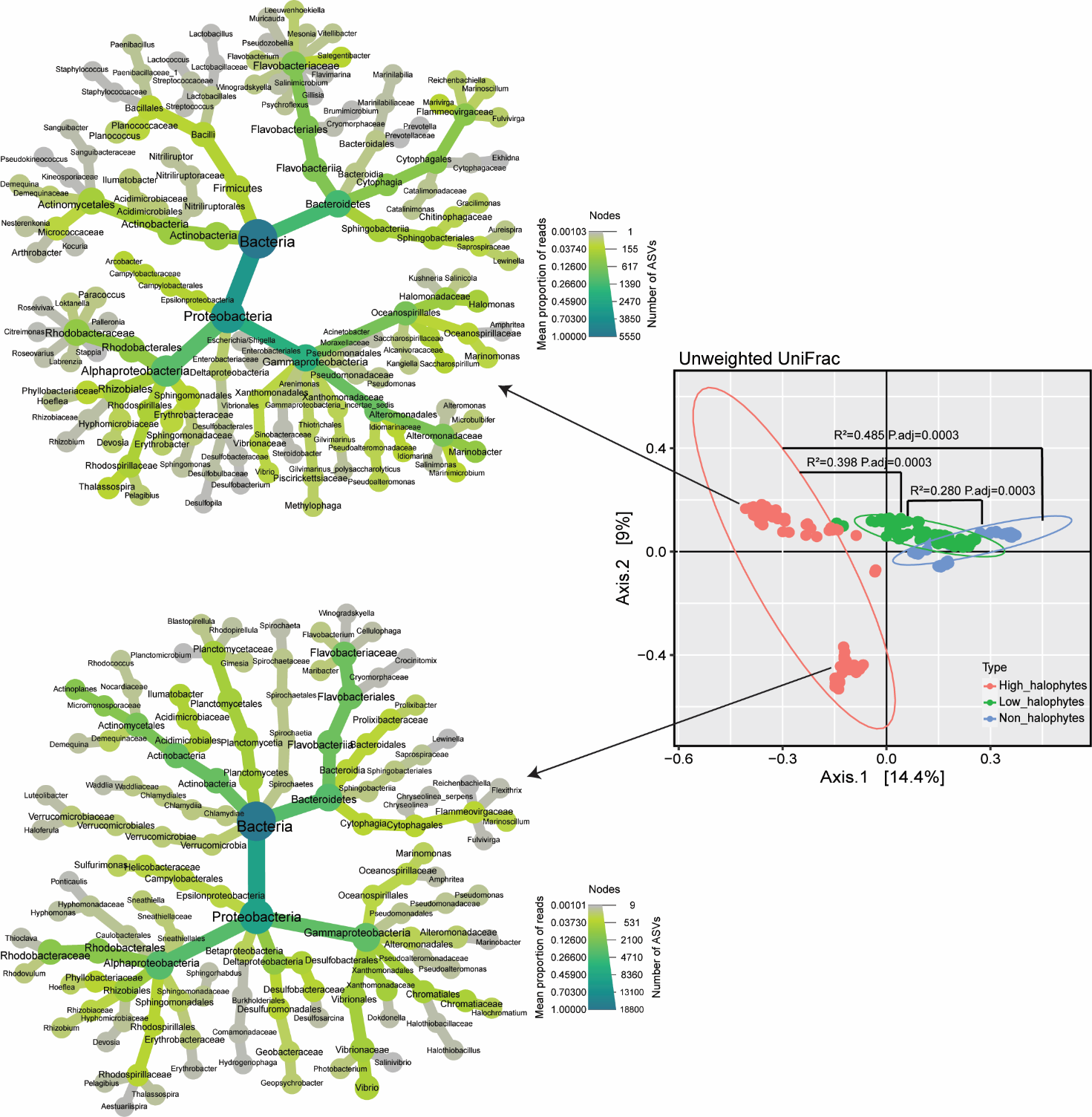


**Figure S2**. According to Figure 1E in the main text for the upper and lower clusters of the high-halophyte group. Heat trees represent the number of ASVs and the read abundance per taxonomic rank from phylum to genus.


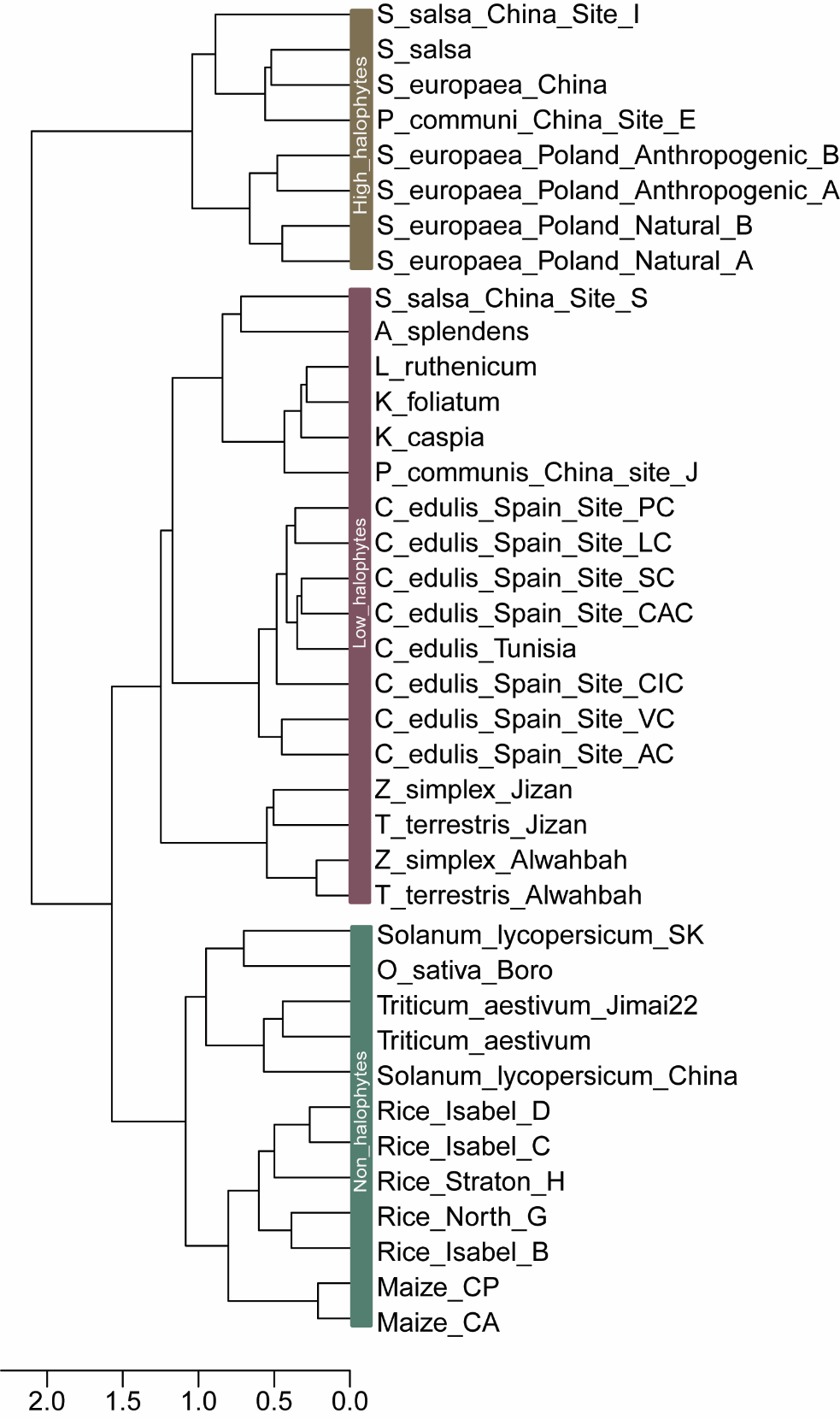


**Figure S3**. Dendrogram based on bacterial genus levels of all plants after removing the outgroup in high-halophytes (bottom cluster).
